# Healthcare users’ evaluations of general practice: a survey among Danish men aged 45–70 years

**DOI:** 10.3399/BJGPO.2024.0153

**Published:** 2025-06-04

**Authors:** Søren Birkeland, Sören Möller

**Affiliations:** 1 Department of Regional Health Research, University of Southern Denmark, and Open Patient Data Explorative Network, Odense University Hospital, Odense, Denmark; 2 Department of Clinical Research, University of Southern Denmark, and Open Patient Data Explorative Network, Odense University Hospital, Odense, Denmark

**Keywords:** Communication, Evaluation, Patient satisfaction, General practice, General practitioners, Primary healthcare

## Abstract

**Background:**

Knowledge about healthcare users’ evaluations of general practice is relatively limited.

**Aim:**

We aimed to investigate Danish men’s evaluations of general practice health care and different aspects of GPs’ communication with patients.

**Design & setting:**

Secondary analyses of data from a web-based survey of 6756 Danish men aged 45–70 years.

**Method:**

We used municipality-level information from registries, self-reported sociodemographic data, personality characteristics, and 5-point Likert scale evaluations of health care and communication in general practice. Comparisons were made between groups using multivariable linear regression.

**Results:**

The response rate was 28%. A large majority of participants agreed or strongly agreed that their GP treatment had been ‘almost perfect’, with slightly fewer responding that their GP was good at showing consideration for them. The latter item was, however, reversed, making comparisons difficult and all differences were small. Older healthcare users evaluated health care higher than did younger healthcare users; higher scores on the extraversion, agreeableness, and conscientiousness personality dimensions generally were associated with higher evaluation scores, whereas the opposite tended to be true for responders with higher neuroticism scores. When not controlling for multiplicity, participants in rural areas tended to evaluate the explanation of medical procedures with lower scores; participants with cerebrovascular disease and those residing in higher tax income areas tended to evaluate GP care less positively in general.

**Conclusion:**

Despite an overall high evaluation of GP care, evaluations may vary, including among different groups of healthcare users.

## How this fits in

Knowledge about healthcare users’ evaluations of general practice is relatively limited. This comprehensive survey among Danish men demonstrated that evaluations were, predominantly, good. More than three in four responders agreed that they received good care; however, evaluations varied slightly across different health care aspects and groups of healthcare users.

## Introduction

Knowledge about healthcare users’ evaluations of general practice health care is relatively limited and much of the existing research dates back in time.^1,2^ Twenty-five years ago, a study comparing satisfaction in various countries reported that healthcare users in Denmark were, generally, among those most satisfied with healthcare services.^3,4^ Correspondingly, in Grol *et al*’s survey study, from the year 2000, conducted among patients in 10 European countries, those visiting their GP were generally very positive about the care provided, with more than 80% viewing it as good or excellent for most of the aspects under study, including GPs’ listening to patients.^2^ In a later Danish cross-sectional study concerning general practice, the vast majority of participants evaluated their GP positively;^5^ in that study, however, participants’ evaluation of various aspects of general practice care varied, including their evaluation of GPs’ communication skills.

Research on the evaluation of GPs by different groups of the population is also limited. A previous nationwide Danish evaluation survey revealed that a positive assessment of GPs was strongly associated with increasing patient age;^6^ however, the association between responders’ gender and the assessment of GPs was weak and inconsistent. In a different study, responders who reported having a chronic condition were more positive in their assessment of GP care than those without a chronic condition.^6^ In a 2010 study, older patients and patients with a chronic illness were more satisfied with GP health care than younger patients and patients without a chronic illness.^7^ More recent studies of patient satisfaction with their GP exist in specific areas of health care; for example, in a 2021 study by Baumbach *et al* featuring a small survey among patients with knee osteoarthritis who were seen in general practice, 59% were satisfied or very satisfied with their knee-related care, and satisfaction was positively associated with greater patient information.^8^

In order to provide higher quality general practice health care, there is a continuous need for knowledge about those aspects to which GPs should give high priority.^5^ For example, less-positive evaluations of specific elements of general practice health care by particular groups of healthcare users may suggest unmet needs in those groups, requiring further clarification and targeted adjustments in practices. In this web-based survey study, we aimed to investigate healthcare users’ evaluations of different aspects of GP care, as well as associations between healthcare user characteristics and their evaluation of GP health care. Given that patient-centeredness has been proven to be an important factor in the evaluation of health care,^2,5,9^ we included an evaluation of GPs’ communication skills in our analyses.

## Method

### Procedures

This is a secondary analysis of separate data from a web-based survey of Danish men aged 45–70 years about medical decision-making regarding prostate disease. Primary analyses aimed to investigate the effect of greater patient involvement in decisions about health care on the evaluation of health care, including information provision. We obtained informed consent from study participants.

Participants were randomised into vignette groups representing different levels of information about disease and healthcare options in a general practice setting. They responded to items, with evaluation of health care illustrated in vignettes and questions about their real-life experiences of healthcare provision. The survey was developed through public and patient involvement. The development and content of the questionnaire is described in detail elsewhere^10^ but, in brief, it included standardised, validated measures (such as the 10-item Big Five Inventory [BFI-10]) as well as purpose-designed items (for example, on participant sociodemographic characteristics).

We drew a random sample of 24000 men aged 45–70 years from the Danish health authorities’ national register of all Danish citizens and, in two waves between January and March 2019, invited them to participate in the study. We used the Research Electronic Data Capture application and administered the survey to our target group through the Danish authorities’ digital mailbox for secure communication with citizens.^11^

### Measures

The analyses reported here concern questionnaire items that are separate from the survey’s vignette element, briefly described above. Information about participants’ sociodemographic characteristics — including civil status, highest completed education, affiliation with the labour market, and chronic illness — was obtained through self-reports. Participants’ personality was measured using the BFI-10, which uses short phrases to assess the most prototypical traits associated with the Big Five dimensions.^12,13^ Regarding chronic illnesses, we referred to the following: cardiovascular disease, diabetes, chronic obstructive pulmonary disease, cerebrovascular disease, cancer, or other. A multimorbidity variable was constructed for participants reporting more than one chronic illness. We also obtained data from the Danish municipalities’ Key Figures database (noegletal.dk) about the sociodemographic characteristics of the participants’ place of residence. These data are a standard measure in Denmark and include statistics about municipality-level population density, tax per citizen, and the proportion of citizens of non-Western origin.

Participants were asked about the medical care they had received from their GP. We, therefore, included purpose-designed questions modified from the 18-item Patient Satisfaction Questionnaire (PSQ-18);^14,15^ these were similar to those in the Danish version of the EUROPEP questionnaire ^16^ (compare doctor–patient relationship, medical care, and information and support domains of the latter instrument). Four questions were included in the outcome definition. First, with explicit reference to ‘the medical care that [the responder] receive[s] from [the responder’s] family physician’, participants were asked to respond to the following statement: *‘*When I seek medical treatment, the doctors are conscientious about examining and treating me’. Afterwards, they were asked to respond to the statements: ‘The medical treatment that I have received has been almost perfect’ and ‘Sometimes doctors ignore what I’m telling them’ (referred to as ‘showing consideration’ when inverted). Finally, participants were asked to respond to the statement: ‘Doctors are good at explaining the cause of medical examinations’. Items were answered on a five-point Likert scale ranging from ‘strongly disagree’ to ‘strongly agree’.

We performed comparisons between groups using multivariable linear regression and reported the mean differences with 95% confidence intervals (CIs). *P* values and 95% CIs were computed by bootstrapping with 1000 repetitions to compensate for residual non-normality. In our main analysis, we reported fully adjusted models, including all sociodemographic factors, and we adjusted for the different randomisation groups (varying level of patient involvement illustrated in vignette) and age. In a sensitivity analysis, we adjusted for randomisation group only. The statistical analyses were performed using Stata (version 18.0), and a *P* value of ≤0.05 was used to indicate statistical significance. We adjusted the statistical tests for multiplicity by using the Holm–Bonferroni method.

## Results

Of the 24000 men invited to participate, 6756 people (28%) completed the survey. Data was, however, missing for one individual and the total amount of responses therefore was 6755. With regard to responders, as described elsewhere, our sample was reasonably representative of men aged 45–70 years in terms of sociodemographic and personality characteristics, when compared with Danish and international data.^17,18^ The average age of the responders was 59.1 years (standard deviation 7.3 years). For the baseline sociodemographic characteristics, see Supplementary Table 1. Table 1 shows participants’ responses when questioned about their experience with the medical care they received from their GP. It appears that responders generally evaluated their doctor’s care well, with three in four, or more, agreeing that they received good care and that their doctor was ‘conscientious about examining and treating them’ and good at explaining medical procedures; slightly less than two-thirds of responders found that doctors were good at ‘showing consideration for them’.

**Table 1.** Participant evaluation of general practice care

Evaluation (Likert ratings)	Component of general practice care, *n* (%)			
	*’When I seek medical treatment, the doctors are conscientious about examining and treating me’*	*’The medical treatment that I have received has been almost perfect’*	*’Sometimes doctors ignore what I’m telling them’ (inverted)*	*‘Doctors are good at explaining the cause of medical examinations’*
Strongly agree	1430 (21)	1653 (24)	845 (13)	1131 (17)
Agree	3969 (59)	4420 (65)	3360 (50)	4010 (59)
Uncertain	684 (10)	377 (6)	976 (14)	1089 (16)
Disagree	606 (9)	268 (4)	1360 (20)	475 (7)
Strongly disagree	66 (1)	37 (1)	214 (3)	50 (1)

The associations between participant characteristics in our model and responders’ assessments of care, according to Likert scale ratings, are shown in Supplementary Table 2. On all the measures, older healthcare users evaluated GP health care slightly better, as shown by a consistent pattern of increasingly positive value coefficients that significantly differed from that of the 45–50-year-old reference group (many findings were significant after implementing a Holm–Bonferroni correction for multiple testing). In addition, patterns appeared to emerge, with lower education being associated with higher scores for doctors’ conscientiousness regarding examination and treatment, as well as doctors’ ability to explain medical procedures; participants’ experiences of GPs’ ability to listen tended to be perceived less favourably (negative coefficients). However, these patterns are not fully consistent, and many of those negative associations were insignificant.

Responders with diabetes experienced greater conscientiousness about examination and treatment, while those with cerebrovascular disease typically rated the quality of GP health care less favorably (a pattern, but with one insignificant association measure). The latter was particularly true for participants’ perception of GPs’ ability to show consideration for them. Responders with higher scores on the extraversion, agreeableness, and conscientiousness dimensions of the Big Five ranked all of the parameters higher (many were also significant after implementing a Holm–Bonferroni correction for multiple testing), whereas the opposite tended to occur for responders with higher scores on the neuroticism dimension. Responders in rural areas tended to evaluate the explanation of medical procedures with lower-ranking scores (a pattern with more insignificant association measures). Lastly, participants residing in areas with lower tax incomes tended to evaluate GPs with higher scores (a pattern with many insignificant association measures).

The sensitivity analysis, adjusted for randomisation group only (Supplementary Table 3), led to very similar results as the main analysis (Supplementary Table 2), with the only clear differences being a significant negative association with BFI-10 neuroticism and conscientiousness results in the sensitivity analysis, no association in the main (fully adjusted) analysis, and positive associations between being retired and multiple outcomes.

## Discussion

### Summary

In this national survey of general practice care in Danish men aged 40–75 years, we found participants generally evaluated health care as good; however, variation persisted in some of the elements of health care experienced by different groups of healthcare users.

### Strengths and limitations

A major strength of this study is the large sample size, and that it is possible to control for many sociodemographic factors.

This study does, however, have many limitations. It was a secondary analysis of data from a larger web-based survey with hypothetical vignettes that focused on patient involvement in medical decision-making about testing for cancer of the prostate, rather than patients’ evaluations of general practice specifically. Furthermore, items were purpose-designed, modified from the PSQ-18, rather than directly taken from, for example, the EUROPEP. Questions such as ‘The medical treatment that I have received has been almost perfect’, like its original wording in the PSQ-18, may have led to bias towards positive feedback;^20^ rephrasing it as an open-ended question — for example: ‘How would you rate the overall quality of medical treatment received?’ — might have decreased bias. Similarly, the statement ‘Doctors are good at explaining the cause of medical examinations’, like its counterpart in the PSQ-18 (and item 12 in the Danish Patients Evaluate General Practice instrument), may not have adequately elicited patients’ perceptions of the health care they received. For example, it would be better to explore whether doctors explain the diagnosis, and address the patient’s ideas, concerns, and expectations. These last aspects provide a better understanding of an effective patient–doctor consultation, whereas explaining the cause of a medical examination is only a small part of the consultation and does not completely reflect its effectiveness. In addition, the inversion of the ‘showing consideration’ item (‘Sometimes doctors ignore what I’m telling them’) may have introduced bias or made comparisons with non-inverted scores more difficult.

The study explicitly focused on general practice health care in Denmark, and we investigated only those evaluations of GPs that were made by men aged 40–75 years. It remains unknown to what extent results may be generalised to other groups of healthcare users. In Heje *et al*’s previous study, there was no general significance of gender with regard to the assessment of GP care.^6^

The 30% response rate, although not uncommon in research using a web-based survey design,^21^ was not optimal. In addition, it raises the question of whether survey participation might have been higher among men who could identify some issues around symptoms of prostate disease and, therefore, whether selection bias was introduced; this cannot be ruled out, even though our sample had been found to be representative of adult men when compared with Danish and international data.

The survey item relating to chronic illness was far from being an accurate representation of the disease burden in Denmark. According to estimations from the Danish National Institute of Public Health, approximately 20% of the Danish population, or 700000–800000 adult Danes, will experience mental health problems during the course of 1 year;^22^ as such, it is clear that mental ill health forms an important part of the disease burden and including perceptions of that population would have provided a more accurate representation of experiences of health care across a wider spectrum of the population.

It should be remembered that Likert ratings reflect evaluations of general practice health care, rather than actual healthcare quality. Previous studies have suggested that correlations between clinical quality and interpersonal aspects of care are low, and that patient experience and clinical outcomes likely represent distinct aspects of healthcare quality.^23,24^ Hence, as suggested by Prang *et al*, patient experience is a valuable marker of patient-centered care and good customer service, rather than a proxy for clinical outcomes.^24^

We used municipality-level information as a proxy for responders’ income, together with self-reported working status. The nested design of this part of the study, including some predictors on a municipality level (although most were on an individual level), could possibly have masked some associations, due to individuals who are uncommon for their place of living (for example, a high-income person living in a low-income municipality) not contributing to possible associations.

Finally, it should be noted that differences in the evaluation of GP care were relatively small and, in many instances, less than 0.2 points on the five-point Likert scale, giving rise to the question of their clinical interpretation and significance. However, given the desire to create higher quality general practice health care through research-based information,^5^ the present findings, regardless of effect sizes, can be considered as one source of data among others to inform the prioritisation of further research and improvement initiatives.

### Comparison with existing literature

A major finding of the present study is that a large majority of healthcare users agreed or strongly agreed that their GP treatment had been “almost perfect”. Even if one could argue that there is still room for improvement, finding about 9 in 10 healthcare users who perceive GP healthcare as almost perfect would seem pleasing to many. Our findings align with results from a cross-sectional study dating from 2008, in which 50774 Danish adults completed the EUROPEP questionnaire and 82.1% responded that their GP’s ‘thoroughness’ was at least ‘good’.^5^ Our findings are also consistent with those of a 2018 survey on patient satisfaction with out-of-hours primary care, in which 82.5% of participants expressed satisfaction with care.^25^ In a later national cluster randomised case–control study among 3609 Danish citizens aged 18 years or older, 66.7% of the responders reported receiving ‘exceptional’ or ‘good’ general practice care during the previous 12 months.^26^ By way of comparison with international findings, in Aljohani *et al*’s survey from 2022, approximately 85% of participants expressed satisfaction with general practice.^27^

We found that GPs were given higher scores by older healthcare users. International studies have previously suggested a relationship between healthcare user age and a more positive evaluation of healthcare.^28–30^ Likewise, in Aljohani *et al*’s survey, being aged >65 years was significantly associated with satisfaction.^27^ As suggested in those previous studies, the association between satisfaction and older age may be rooted in: a patient’s longstanding relationship with their GP; the patient having a more realistic view of health and health care, expectations, and doctors’ skills due to their own life experience; and, perhaps, older people having a more forgiving way of judging — being a ‘grateful generation’ — with regard to health care. In a nationwide Danish patient evaluation survey that included 28260 patient evaluations, a positive GP assessment was found to be strongly associated with increasing patient age and increasing frequency of attendance;^6^ furthermore, patients reporting a chronic condition were more likely to be positive. Our study adds nuances to those previous findings by suggesting that evaluations of general practice care may vary among patients with different chronic illnesses. Contrary to our findings, in that same study, no association was found between a positive GP assessment and patients’ education level.^6^

Finally, we found that healthcare users in areas with higher tax incomes assessed GP care more critically. These findings echo those of previous international research that found lower levels of satisfaction among patients who were affluent.^9,31^ As previously suggested, there may be socioeconomic differences in health service expectations, as people with more socioeconomic resources may expect their doctor to communicate particularly well and provide clear information.^9,32^

In a Norwegian survey from 2017, responders found, to a large extent, that GPs were showing interest in their situation, providing sufficient information, and including them as much as they would like in decisions.^33^ In our study, more than three-quarters of healthcare users reported that their GPs’ skills in explaining medical procedures were good, while slightly less than two-thirds of responders thought their GPs were good at listening and showing consideration for them. Although the reasons for this finding are unknown, a tentative explanation could be that high workload and signs of burnout are common among GPs,^34^ and stress and burnout may affect listening skills, as well as the competency to show consideration for patients, be attentive, and listen to others.^35,36^

In contrast with previous findings from Denmark^5^, the evaluation of GPs’ skills in terms of showing consideration for their patients in the present study was least positive. Furthermore, in our study, we found that responders in urban areas tended to evaluate the explanation of medical procedures as better. By way of comparison, in Iqbal *et al*’s 2021-study from Scotland, individuals residing in remote and rural areas tended to have the highest satisfaction with their general practice in terms of patient-centered care.^9^

### Implications for research and practice

To continuously achieve the best possible quality in general practice, knowledge is needed about where, in particular, to prioritise improvement efforts.^5^ The findings suggested persisting inequalities in aspects of GP care that may need to be addressed, as well as the importance of doctors’ skills in showing consideration for their patients, informing them, and providing patient-centered care. Studies are needed to both deepen our understanding of healthcare users’ views on GP care and validate the findings of the present analyses. As an example, our finding of an age gradient in healthcare users’ evaluation of GP care should be followed up to clarify to what extent this mirrors the evolvement of a more demanding population of healthcare users that necessitates change at GP level (for example, relating to further education or training) or at higher organisational levels that requires intensified information to accommodate the expectations of younger healthcare users.

Our findings that suggested that participants with cerebrovascular disease have a slightly less positive evaluation of GP care also merits further investigation to verify whether this reflects a common experience in this group of citizens and, if so, to better meet the needs of individuals with cerebrovascular disease in general practice. In the same way, studies are needed to clarify healthcare users’ evaluations of mental health care in general practice.

In future studies, more accurate financial status should also be collected; this could be done directly from responders or through linkage with tax record registries. Last, but not least, in many countries, women face unique challenges in accessing health care, and their satisfaction levels may differ significantly from those of men. As such, including women in future studies would help to identify any gender-specific issues and provide a more comprehensive understanding of healthcare perceptions across the entire Danish population, thereby ensuring that healthcare services accommodate the needs of all patients. Research findings may then inform the design of means to improve the delivery of general practice services. Improvement initiatives should also include measures of clinical outcomes and patient satisfaction to obtain a comprehensive evaluation of care.

The findings of this study suggested that, in spite of GP care being evaluated highly overall, evaluations vary among healthcare users with different sociodemographic backgrounds, personality styles, and health problems; this underscores the need for GPs to be attentive to patients’ different needs, views, and concerns.

## Data

The dataset relied on in this article is available from the corresponding author on reasonable request.
